# Exploiting distant homologues for phasing through the generation of compact fragments, local fold refinement and partial solution combination

**DOI:** 10.1107/S2059798318001365

**Published:** 2018-04-03

**Authors:** Claudia Millán, Massimo Domenico Sammito, Airlie J. McCoy, Andrey F. Ziem Nascimento, Giovanna Petrillo, Robert D. Oeffner, Teresa Domínguez-Gil, Juan A. Hermoso, Randy J. Read, Isabel Usón

**Affiliations:** a Crystallographic Methods, Institute of Molecular Biology of Barcelona (IBMB–CSIC), Barcelona Science Park, Helix Building, Baldiri Reixac 15, 08028 Barcelona, Spain; b Department of Structural Chemistry, Georg August University of Göttingen, Tammannstrasse 4, 37077 Göttingen, Germany; c Cambridge Institute for Medical Research, University of Cambridge, Hills Road, Cambridge CB2 OXY, England; d XALOC Beamline, Experiments Division, ALBA Synchrotron Light Source, Cerdanyola del Vallès, 08290 Barcelona, Spain; e Brazilian Synchrotron Light Laboratory (LNLS), Brazilian Center for Research in Energy and Materials (CNPEM), Caixa Postal 6192, 13083-970 Campinas-SP, Brazil; f Biochemize S.L, Barcelona Advanced Industry, C/Marie Curie 8-14, 08042 Barcelona, Spain; g Department of Crystallography and Structural Biology, Instituto Química-Física ‘Rocasolano’ CSIC (Spanish National Research Council), Serrano 119, 28006 Madrid, Spain; h ICREA, Institució Catalana de Recerca i Estudis Avançats, Passeig Lluís Companys 23, 08003 Barcelona, Spain

**Keywords:** phasing, *ARCIMBOLDO_SHREDDER*, small fragments, molecular replacement, fragment-based molecular replacement

## Abstract

Macromolecular structures can be solved by molecular replacement provided that suitable search models are available. Models from distant homologues may deviate too much from the target structure to succeed, notwithstanding an overall similar fold or even their featuring areas of very close geometry. Successful methods to make the most of such templates usually rely on the degree of conservation to select and improve search models. *ARCIMBOLDO_SHREDDER* uses fragments derived from distant homologues in a brute-force approach driven by the experimental data, instead of by sequence similarity. The new algorithms implemented in *ARCIMBOLDO_SHREDDER* are described in detail, illustrating its characteristic aspects in the solution of new and test structures. In an advance from the previously published algorithm, which was based on omitting or extracting contiguous polypeptide spans, model generation now uses three-dimensional volumes respecting structural units. The optimal fragment size is estimated from the expected log-likelihood gain (LLG) values computed assuming that a substructure can be found with a level of accuracy near that required for successful extension of the structure, typically below 0.6 Å root-mean-square deviation (r.m.s.d.) from the target. Better sampling is attempted through model trimming or decomposition into rigid groups and optimization through *Phaser*’s *gyre* refinement. Also, after model translation, packing filtering and refinement, models are either disassembled into predetermined rigid groups and refined (*gimble* refinement) or *Phaser*’s LLG-guided pruning is used to trim the model of residues that are not contributing signal to the LLG at the target r.m.s.d. value. Phase combination among consistent partial solutions is performed in reciprocal space with *ALIXE*. Finally, density modification and main-chain autotracing in *SHELXE* serve to expand to the full structure and identify successful solutions. The performance on test data and the solution of new structures are described.

## Abstract 2

*ARCIMBOLDO_SHREDDER* solves structures using fragments from low-homology models. Search fragments are improved through refinement or trimming against the experimental data. Consistent solutions are combined.

## 1. Introduction   

The successful use of distant homologues as search models for molecular replacement (MR) often requires the initial template to undergo a significant degree of improvement as, notwithstanding the overall correct fold or their featuring areas of close geometry, differences may prevent a solution. Model improvement can be contrived by relying on the degree of conservation as implemented in *Sculptor* (Bunkóczi & Read, 2011 ▸), combining a range of models (Leahy *et al.*, 1992 ▸) as in *Ensembler* (Bunkóczi *et al.*, 2013 ▸), sampling model deformation along normal modes (McCoy *et al.*, 2013 ▸; Suhre & Sanejouand, 2004 ▸) or modelling within protocols devised for this purpose in *Rosetta* (DiMaio *et al.*, 2011 ▸), *QUARK* (Xu & Zhang, 2012 ▸) or *I-TASSER* (Zhang, 2008 ▸). Fragmenting and reassembling search models has also been explored (Shrestha & Zhang, 2015 ▸).

Methods exploiting the combination of molecular replacement using partial models or fragments with density modification and automated map interpretation may bootstrap to a full solution even if only a small fraction of the asymmetric unit content is placed by MR (Yao *et al.*, 2006 ▸). Programs such as *ARCIMBOLDO* (Rodríguez *et al.*, 2009 ▸) and *AMPLE* (Bibby *et al.*, 2012 ▸) rely on *Phaser* (McCoy *et al.*, 2007 ▸), in particular the rotation (Storoni *et al.*, 2004 ▸) and translation functions (McCoy *et al.*, 2005 ▸), to place the fragments, and on *SHELXE* to apply density modification (Sheldrick, 2002 ▸) and to extend the very incomplete solutions into an interpretable trace (Sheldrick, 2010 ▸).

In *ARCIMBOLDO_SHREDDER* (Sammito *et al.*, 2014 ▸), fragments are derived from a distant homologue template, and their performance is jointly evaluated in a process driven by the experimental data, provided that a resolution of at least 2.5 Å is available. In its original implementation, template trimming relied on a rotation-function-based scoring: the SHRED-LLG. The whole template was initially used to find the maxima of the rotation function. The list of peaks in the rotation function was clustered within a given tolerance. For each of these clusters, the template was systematically shredded (omitting continuous stretches with a range of sizes from the polypeptide chain) and the fragments were scored against each unique solution of the rotation function. The results were then combined into a score per residue and the template was trimmed accordingly. The sequential shredding and its derived model trimming can improve models where the high average deviation from the target is owing to dissimilar or flexible regions reducing the signal from a core of low root-mean-square deviation from the target structure (r.m.s.d.).

An assumed RMSD value is a key parameter in the likelihood calculations, determining the relative weights assigned to low- and high-resolution data (here, RMSD is used to describe the parameter value, to distinguish it from the actual deviation from the final structure, which is denoted r.m.s.d.). Assigning an optimal value for a particular model will yield the highest LLG scores and the best signal to noise in a search with that model (Oeffner *et al.*, 2013 ▸). However, in the context of *ARCIMBOLDO* the requirement is to obtain models that are highly accurate, even at the expense of completeness, because the model-completion step only succeeds when the models have overall r.m.s.d. values below 0.6 Å. Therefore, the goal is to select, from many possible models, models that will provide this level of accuracy, a selection assisted by setting the corresponding target RMSD. Because models can be improved before completion (by the *gyre*, *gimble* and pruning steps described in more detail below), the initial search can use a somewhat higher target RMSD, which is gradually reduced throughout the model-improvement steps. Suitable initial values depend on the size of the problem, but can vary from 0.5 to 2.0 Å.

Here, we present a new implementation of the *ARCIMBOLDO_SHREDDER* algorithm extended to use fragments defining an approximately spherical volume in order to extract and improve compact structural units from an initial low-identity template. The original implementation of this idea, which aimed to eliminate all the most incorrect regions in the starting model, has been further extended to correct them through refinement. Partial models, sometimes comprising as little as 10% of the main-chain atoms in the asymmetric unit, need to be very accurate (r.m.s.d. of around 0.6 Å) for their correct placement and extension into the full structure at 2 Å resolution. In order to increase the radius of convergence of this approach, additional degrees of freedom are given to the models, which are decomposed and subjected to refinement against the intensity-based likelihood rotation-function target (RF; Read & McCoy, 2016 ▸) and again after they have been placed in the unit cell. This refinement is accomplished in *Phaser* with the *gyre* and *gimble* modes (McCoy *et al.*, 2018 ▸). The use of the *ARCIMBOLDO_SHREDDER* spheres mode on test structures as well as in the solution of previously unknown structures is illustrated.

## 2. Materials and methods/experimental   

### 2.1. Computing setup   

Structure solutions and tests were run on a local HTCondor v.8.4.5 (Tannenbaum *et al.*, 2001 ▸) grid made up of 160 nodes totaling 225 GFlops. Submitter machines were eight-core workstations with 24 GB RAM running Debian or Ubuntu Linux. The typical running times on the grid for the cases described in this paper are 2–19 h, but timing is approximate as grid access was shared with other users.

### 2.2. Software versions   

The *ARCIMBOLDO_SHREDDER* binary is deployed for Linux and Macintosh current OS (Mavericks to Sierra 10.12.1). It is generated with PyInstaller 3.3 and Python 2.7.12. The experiments described in this study relied on *SHELXE* versions from 2016 onwards and *Phaser* versions from 2.7.*x* onwards. The figures of merit used in decision making were *Phaser*’s intensity-based log-likelihood gain (LLG; Read & McCoy, 2016 ▸) and the correlation coefficient between observed and calculated normalized intensities (CC; Fujinaga & Read, 1987 ▸) calculated by *SHELXE* (Sheldrick, 2002 ▸). Structure-amplitude-weighted mean phase errors (wMPE; Lunin & Woolfson, 1993 ▸) were calculated with *SHELXE* against the models available from the PDB to assess performance.

The model and maps were examined with *Coot* (Emsley *et al.*, 2010 ▸). Figures were prepared with *PyMOL* (v.1.8; Schrodinger). Tutorials and documentation are available from our website (http://chango.ibmb.csic.es/SHREDDER).

### 2.3. New structures and test data   

The characteristics of all data used in this study are summarized below and relevant statistics are given in Table 1 ▸. The set revisits structures first solved using prototypes of the present implementation and includes additional tests with other folds. In most cases correct intermediate solutions are scarce, which shows their difficulty but also hinders systematic testing. The mainly helical structure LTG is the only one where many partial solutions are produced, which allows the effect of parameterization to be probed.

#### 2.3.1. Novel structure LTG   

LTG is a soluble lytic transglycosylase from *Pseudomonas aeruginosa* (PDB entry 5ohu, unpublished work). Diffraction data collected at the ALBA synchrotron to 2.1 Å resolution were available. The crystals belonged to space group *P*6_3_, with unit-cell parameters *a* = *b* = 163.98, *c* = 56.71 Å. The asymmetric unit contains a monomer of 613 residues of the mainly helical structure, along with 61% solvent.

#### 2.3.2. Novel structure Hhed2   

Hhed2 is a halohydrin dehalogenase from a gammaproteobacterium (Schallmey *et al.*, 2014 ▸; Koopmeiners *et al.*, 2017 ▸). Diffraction data collected at the ALBA synchrotron to 1.6 Å resolution were available. The crystals belonged to space group *P*2_1_2_1_2_1_, with unit-cell parameters *a* = 78.02, *b* = 94.86, *c* = 140.27 Å. The asymmetric unit contains four copies of a monomer, totalling 922 residues, along with 50% solvent content.

#### 2.3.3. Novel structure PPAD   

PPAD is a peptidylarginine deiminase from *P. gingivalis* (Goulas *et al.*, 2015 ▸). 20 diffraction data sets from different crystals were available, ranging from 2.97 to 1.5 Å resolution. 16 of these, with unit cells of similar dimensions and rendering an average *R*_int_ of 0.37 and *R*_σ_ of 0.02, were combined. The crystals belonged to space group *P*2_1_2_1_2_1_ and contain one copy of the 432-amino-acid monomer in the asymmetric unit, corresponding to a solvent content of 40%, which was set to 50% in *SHELXE* runs to account for possible disordered regions. The structure features short helices and twisted β-sheets along with a high proportion of coil.

#### 2.3.4. Test case 1yzf   

PDB entry 1yzf is a lipase/acyl­hydrolase from *Enterococcus faecalis* (Midwest Center for Structural Genomics, unpublished work). The structure shows a central β-sheet flanked by helices. Data to 1.9 Å resolution are available from the PDB from crystals belonging to space group *P*3_2_21, with unit-cell parameters *a* = *b* = 45.92, *c* = 148.03 Å. There is one monomer totalling 195 residues in the asymmetric unit, corresponding to a low solvent content of 36%.

#### 2.3.5. Test case 3fp2   

PDB entry 3fp2 is the crystal structure of Tom71 in complex with a C-terminal fragment of Hsp82 (Li *et al.*, 2009 ▸). Data to 2.0 Å resolution are available from the PDB from crystals belonging to space group *P*2_1_2_1_2_1_, with unit-cell parameters *a* = 47.86, *b* = 116.29, *c* = 150.74 Å. There is one monomer of Tom71 of 537 residues plus a 12-residue fragment of the ATP-dependent molecular chaperone HSP82, totalling 549 residues, in the asymmetric unit, corresponding to a solvent content of 63%. The structure is mainly helical.

## 3. Results and discussion   

### 3.1. *ARCIMBOLDO_SHREDDER* spherical mode   

**Figure 1:**
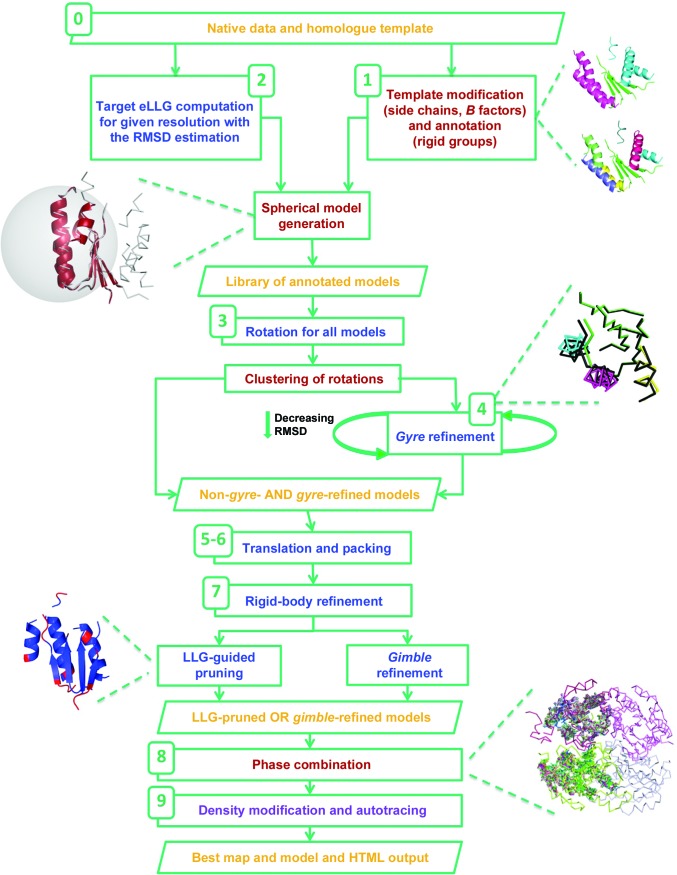
*ARCIMBOLDO_SHREDDER* workflow. The numbers reference the steps described in §[Sec sec3.1]3.1. Orange colour refers to input/output, blue to *Phaser* steps, red to *ARCIMBOLDO* steps and purple to *SHELXE* steps.

Fig. 1 ▸ summarizes the program flow of *ARCIMBOLDO_SHREDDER* and Table 2 ▸ describes all operations to modify the search models throughout the program flow. The grid computing implementation is described in Appendix *A* [App appa].

The program accepts a configuration file, with extension .bor, which contains the parameterization of the run. Most parameters have appropriate defaults, and the only mandatory input is the data description, a template model and the shredding mode. The generation and evaluation of sequentially shredded models is mostly unchanged from the algorithm described in 2014 (Sammito *et al.*, 2014 ▸), as reviewed in §[Sec sec1]1. In this paper, the spherical mode shredding by volume and structure is described.

*Step 0. Initial checks*. The first task performed by the program is validation of the instruction file, which must contain all mandatory parameters and may override defaults. Non-existent or misspelt instructions will be ignored and physically impossible values, such as a negative value for the molecular weight, or a model size larger than the given template will cause the program to terminate. Further checks to ensure the run is viable comprise validation of paths to files and folders, format correctness of the input files, retrieval of hardware information and the compatibility of *Phaser* and *SHELXE* versions. The resolution of the data is also checked and if it is below 2.5 Å the run will be terminated.
*Step 1. Partitioning and annotation of the template*. The template model is pre-processed, analysed and annotated in terms of fragments that will be treated as rigid groups in *gyre* and *gimble* refinement. The default pre-processing trims side chains and sets a common *B* value for all atoms. The user can override either default to preserve this information in the template model. The secondary-structure elements that are present in the model are identified relying on the distribution, distances and angles between characteristic vectors defined from the centroids of C^α^ atoms to the centroids of carbonyl O atoms from all tripeptides. The relations among characteristic vectors also allow characterization of the tertiary structure (Sammito *et al.*, 2013 ▸). Unless otherwise selected, coil regions in the template are trimmed. A first level of annotation partitions the secondary-structure elements into a few groups defined by distance and the preservation of folds such as association of strands into a sheet. A second level further separates individual helices. This partition scheme is established on the template using community clustering (Clauset *et al.*, 2004 ▸; Csárdi & Nepusz, 2006 ▸; Pons & Latapy, 2005 ▸; Rosvall *et al.*, 2009 ▸) and tertiary-structure constraints, and is adopted for each of the partial models derived. Chain identifiers are used to mark rigid groups. By default, they are set and modified by the program in the course of the *ARCIMBOLDO_SHREDDER* run as the fragment is progressively decomposed into more rigid bodies. Alternatively, the user may input a template that is already annotated with chain identifiers and set the program to preserve them.
*Step 2. Generation of the models*. After the template has been annotated for partition, a library of equal-sized models is generated. The expected LLG (eLLG; McCoy *et al.*, 2017 ▸) provides an estimate of the model size that is required to identify correct solutions for a particular molecular-replacement problem. Its value depends on the accuracy and completeness of the model, and on the number of reflections. The *Phaser* MR_ELLG mode is thus used to estimate the number of polyalanine residues needed in order to reach a target eLLG for the available data assuming an RMSD value. Models are generated to fit the calculated size. The eLLG defaults used within *ARCIMBOLDO_SHREDDER* are somewhat at the lower limits compared with a general molecular-replacement case since as long as nonrandom solutions are generated, combination of partial solutions and subsequent density modification and autotracing will discriminate the correct solutions. The computation of the expected LLG is performed even if the user sets the model size, and the program will issue a warning if the chosen parameterization appears to be unfavourable.The previously described sequential shredding mode is still available (Sammito *et al.*, 2014 ▸). In this mode, fragments of different sizes are systematically omitted from the template to simultaneously identify all of the most incorrect regions. Conversely, the spherical mode provides a way to cut models in a spatial way, retrieving compact fragments that are structurally close rather than contiguous in sequence. This is performed by traversing the sequence and using each residue in turn as the centre of a sphere containing the number of residues estimated from the eLLG. For each model, residues are selected by their distance to the central amino acid, subject to the constraints of preserving secondary-structure continuity and avoiding unconnected stretches of less than four residues for strands or seven for helices. All models are gathered in a library.In subsequent steps the library is used and evaluated with an algorithm similar to that previously described for *ARCIMBOLDO_BORGES* (Millán, Sammito & Usón, 2015 ▸) although the parameterization and options are specifically devised for *ARCIMBOLDO_SHREDDER*. In contrast, the models derived from homologues in the sequential mode undergo a subsequent *ARCIMBOLDO_LITE*-like treatment. *ARCIMBOLDO_BORGES* was originally designed to evaluate libraries of superimposed local folds of the same size, such as three β-stranded antiparallel β-sheets, extracted from the PDB (Berman *et al.*, 2000 ▸). The common size ensures that the figures of merit are comparable and, given the superposition of the initial models, equivalent rotations bring models to the same position.
*Step 3. Evaluation against the likelihood rotation-function target*. An independent rotation search is performed on each of the models in the library. The resulting rotation angles are clustered within a given threshold (15° by default), taking symmetry into account, and all models producing rotations in the same cluster are gathered. A model usually populates more than one cluster, either because the asymmetric unit contains more than one copy of the structure, because small fragments may fit different parts of a structure or because incorrect solutions are obtained along with correct ones. In either case, it is convenient to isolate these different situations, so that from this point on every step is performed independently on each rotation cluster. Also, subsequent default filters are used independently unless a given cluster is aborted, so that diversity is preserved while keeping the number of solutions within manageable limits.
*Step 4. *Gyre* refinement*. Models can be subject to a step of *gyre* refinement (McCoy *et al.*, 2018 ▸) against the intensity likelihood rotation target (Read & McCoy, 2016 ▸) starting at their highest scoring rotation solution for the given cluster. Atoms with different chain identifiers within an ensemble will be treated as independent rigid groups, refining their rotation and relative translation. In *gyre* refinement, an initial RMSD parameter is chosen as a tradeoff between convergence radius and sensitivity to coordinate accuracy, iterating refinement and decreasing the RMSD parameter estimation sequentially. The goal is to improve and select among the many possible models those with a true r.m.s.d. of below 0.6 Å, and thus susceptible of being expanded to the full solution in the density-modification and autrotracing step.The chain definition also changes between cycles in order to increase the number of fragments and thus the degrees of freedom for model refinement, as predefined in the template-partitioning step (step 1).
*Step 5. Translation search*. Both rotated and *gyre*-refined models in each cluster are subjected to a translation search. The RMSD value of the last cycle of *gyre* refinement will be used for the translation search and all subsequent steps until VRMS refinement for both *gyre*-refined and non-*gyre*-refined models.
*Step 6. Packing test*. Translated solutions are filtered with the *Phaser* packing function. In *ARCIMBOLDO_SHREDDER*, as the models tend to be larger and are expected to be less accurate, the default for the packing test allows 3% clashes instead of the very stringent default in the other *ARCIMBOLDO* modes, which accepts no clashes.
*Step 7. Refinement*. *Phaser*’s rigid-body refinement is performed on all solutions accepted by the packing test. If refinement of the variance-r.m.s. parameter (VRMS; Oeffner *et al.*, 2013 ▸) has been set, it will be performed at this stage. Optionally, the original template may be superimposed on each placed fragment, and trimming and refinement of the model is revisited. Whether on the small, placed fragments or on the whole template, two different methods of optimization are available. A *gimble* (McCoy *et al.*, 2018 ▸) refinement step, subdividing the placed model into the same rigid groups as differentiated in *gyre*, can be subsequently applied. Alternatively, *Phaser*’s likelihood-based pruning can be used to eliminate from the refined model those residues whose removal leads to an increase in the LLG (Oeffner *et al.*, 2018 ▸). The RMSD set at the pruning step will determine the trade-off between completeness and accuracy in the resulting model.
*Step 8. Phase combination*. Solutions from both the original and the refined models are passed to *SHELXE* to compute the initial correlation coefficient (CC) and for five cycles of density modification. This leads to some discrimination between protein and solvent regions. This is possibly the reason why even for phase sets with mean phase errors that are too large to be improved, determination of the relative origin shift is enhanced. The phase sets produced are sorted according to their figures of merit (CC, LLG at refinement and TFZ score). At this point, consistent phase sets can be combined in order to complete partial solutions and increase their information content. This is performed within *ARCIMBOLDO_SHREDDER* by an integrated version of *ALIXE* (Millán, Sammito, García-Ferrer *et al.*, 2015 ▸), using a two-step procedure. Firstly, for each rotation cluster partially overlapping solutions are identified within 60° mean phase difference to the clustered phases. Subsequently, if the asymmetric unit is expected to contain more than one monomer, a second round combines phase sets gathered in the first step from different rotation clusters, allowing a higher tolerance (87°).
*Step 9. Density modification and autotracing for expansion of the substructure to a full solution*. The single or combined phase sets are used to calculate starting maps for iterative density modification and autotracing with *SHELXE*. If phase combination is disabled or the combined phases do not yield a solution, the procedure is performed on selected individual solutions.The figures of merit used for selection depend on the previous steps: CC after having performed a correlation CC-guided trimming (-o) in *SHELXE*, or LLG otherwise. In either case, solutions characterized by top CC, LLG and TFZ score will be included in the selected set.
*Step 10. Best-solution traceback and output of figures of merit*. Throughout the run, an HTML output that is generated at the beginning is continually updated with the figures of merit corresponding to each of the steps. While density modification and autotracing is being performed in *SHELXE*, the trace with the highest CC is highlighted at every cycle in the HTML. Values above 30% typically indicate a solved structure at a resolution better than 2.5 Å (Usón & Sheldrick, 2018 ▸). When the program finishes, the HTML output file describes the best solution found and its figures of merit, together with links to its map and coordinate files.

### 3.2. Solution of an all-helical previously unknown structure: LTG   

**Figure 2:**
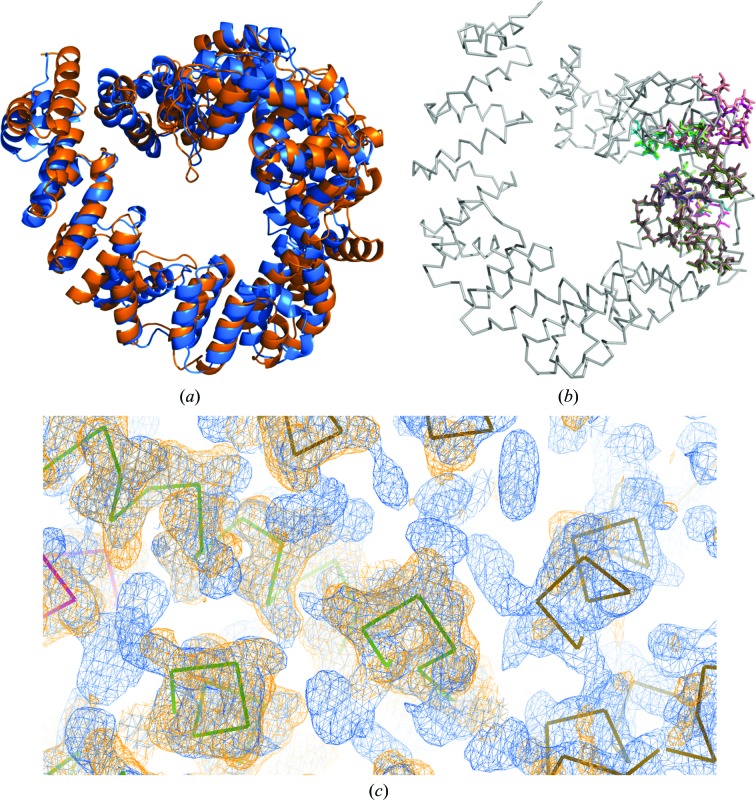
Original solution of LTG. (*a*) Final structure (blue) *versus* the template used in *ARCIMBOLDO_SHREDDER* (orange). The r.m.s.d. between the structures is 4.6 Å over a core of 582 C^α^ atoms. (*b*) Coloured sticks show the solving fragments that clustered together and the black ribbon shows the final structure. (*c*) A detail of the *SHELXE* *F*_o_·FOM electron-density maps with the C^α^ trace. Orange, initial map from phase combination; blue, final map after density modification and autotracing; both are contoured at 1σ.

LTG is a soluble lytic transglycosylase from *P. aeruginosa*. Data sets were collected on the XALOC beamline at ALBA (Juanhuix *et al.*, 2014 ▸). A homology search for the target sequence using *HHpred* (Söding *et al.*, 2005 ▸) provided a list of possible templates for molecular replacement. The best-scoring model was another soluble lytic transglycosylase, SLT70 from *Escherichia coli* (PDB entry 1qsa; van Asselt *et al.*, 1999 ▸), with 31% sequence identity. The estimated VRMS for this degree of conservation is 1.5 Å, but on account of its flexibility the r.m.s.d. of the final structure with respect to the 1qsa model is 4.6 Å, as computed with the *PyMOL* super algorithm on a core of 582 residues. Fig. 2 ▸ shows the superposition of the final structure and template (Fig. 2 ▸ *a*), the fragments used in the solution (Fig. 2 ▸ *b*) and a detail of the electron-density maps before and after expansion (Fig. 2 ▸ *c*).

The structure was originally solved with *ARCIMBOLDO_SHREDDER* in the first implementation of the spherical mode, which is less developed than that currently released and described here. The full PDB structure of 1qsa was used as the initial template, preserving the coil regions and the original *B* factors, but trimming the side chains to alanines. Spheres of 20 Å radius centred on each amino acid of the template were defined, without further modification, to extract 619 models. Those models ranged in size from 42 to 177 residues, making the figures of merit not directly comparable across fragments. It should be stressed that all models are naturally superimposed on the template that they derive from and correspond to different parts of a common fold. Therefore, they can be input as a library into *ARCIMBOLDO_BORGES*. Similar rotations would map fragments to consistent regions of the target structure if the original fold were maintained. Moreover, partially overlapping solutions, if produced, should be found within one rotation cluster and their maps could be combined to improve the starting phases. In this case, one of the rotation clusters stood out through solutions with TFZ scores above 8. Such solutions were used as references to cluster phases. One of the combined phase sets developed into a full solution, with a CC of 48.08% and 563 residues traced in seven chains. All 12 models thus grouped were targeting the same region of the query structure, corresponding to residues 478–592 in the template.

### 3.3. Solution of a previously unknown structure: PPAD   

The structure of the peptidylarginine deaminase from *P. gingivalis* was originally solved by manually generating fragments from up to six different homologous templates, ranging in sequence identity from 22 to 18%, and using them as search fragments in *ARCIMBOLDO* runs (Goulas *et al.*, 2015 ▸). The common fold in all of these structures is a pentein β/α propeller composed of five α–β–β–α–β units arranged around a pseudo-fivefold axis. One of the models cut out from the 1zbr template (a template with 19% sequence identity and an r.m.s.d. of 1.5 Å over a core of 231 C^α^ atoms), composed of the polyalanine-trimmed fifth and first repeats, stood out in one of the many parameterizations tested. This case produced a single rotation cluster and a lower number of solutions with a higher LLG than any other trial or model. A resolution cutoff of 2.1 Å was used for the RF, a resolution cutoff of 1.7 Å was used for the translation search and the RMSD was set to 0.8 Å. Still, its expansion did not yield a solution. Using this solution as a reference, phase clustering identified a consistent solution coming from a partially overlapping model and their combination was successfully expanded.

### 3.4. Solution of a previously unknown structure from an α/β enzyme: Hhed2   

Hhed2 is a 230-amino-acid halohydrin dehalogenase from a gammaproteobacterium. Data to a resolution of 1.6 Å were available from crystals belonging to space group *P*2_1_2_1_2_1_, with four monomers in the asymmetric unit totalling 920 residues. A homology search for the target sequence using *HHpred* provided a list of possible templates for molecular replacement, sharing a typical Rossmann fold characterized by a series of alternating β-strand and α-helical segments with the β-strands arranged in a parallel β-sheet.

Three homologues were selected, two of which were from the same family of dehalogenases, HhedB (PDB entry 4zd6) with a sequence identity of 47% and HheA (PDB entry 4z9f) with a sequence identity of 30% (Watanabe *et al.*, 2015 ▸), and one of which was from the same superfamily of short-chain dehydrogenase reductases (SDRs), EbN1 (Büsing *et al.*, 2015 ▸) with 26% sequence identity.

**Figure 3:**
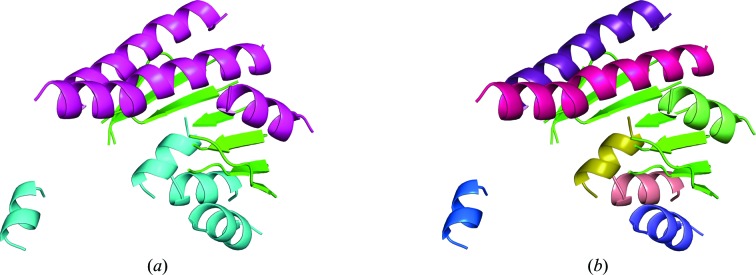
Annotation levels for the 4urf model. (*a*) First-level annotation groups. (*b*) Second-level annotation separating the β-sheet and independent helices.

All three templates lead to a successful solution as shown in Table 3 ▸. The two dehalogenases show r.m.s.d.s to the target structure over a core of 185 C^α^ atoms of 0.7 Å (PDB entry 4z9f) and 1.12 Å (PDB entry 4zd6), respectively; for the SDR (PDB entry 4urf) the r.m.s.d. over a core of 149 C^α^ atoms is 1.3 Å. The templates were trimmed, removing short α-helices of less than seven residues, β-strands of less than four residues and coil regions. The annotation for the first *gyre* cycle leaves the central β-sheet present in the fold as a single, indivisible group. A second level of annotations separated the helices as independent groups. Figs. 3 ▸(*a*) and 3 ▸(*b*) show both levels of annotation for PDB entry 4urf which are consistent with those of PDB entries 4zd6 and 4z9f. In all cases, the rotation search and the first cycle of *gyre* refinement were performed at 0.8 Å RMSD. A second cycle of *gyre* refinement and subsequent *Phaser* steps were performed at 0.5 Å RMSD. The size of the search fragments was set in order to achieve a target eLLG of 60 at the last RMSD used in the run (0.5 Å). All relevant parameters and results are described in Table 3 ▸.

#### 3.4.1. Template 4zd6   

The template derived from PDB entry 4zd6 is so close to the target structure that solution is trivial. Fragments derived from this model are correctly placed corresponding to all four monomers in the asymmetric unit, although approximate alignment of noncrystallographic and crystallographic symmetry axes leads to three, rather than four, rotation clusters. All best-scoring fragments have been improved by *gyre* and *gimble*. Consistent solutions were combined using the best-scoring solution, characterized by a TFZ score of 12.6, as a reference. Two consecutive combination steps setting mean phase difference thresholds of 60 and 87° identify the remaining correct solutions placed on the same and different monomers, respectively.

This phase set, when submitted to *SHELXE* for density modification and autotracing, solves the structure and reaches a CC of 37.99%, with 859 residues traced in 13 chains.

#### 3.4.2. Template 4z9f   

The template derived from PDB entry 4z9f also gives rise to two rotation clusters containing correct solutions and characterized by final LLG values clearly discriminating them from the remaining clusters (133 and 129 *versus* 99). As seen from Table 3 ▸, the number of correct partial solutions is markedly lower than with the previous template. *Gyre* and *gimble* model refinement improves the wMPE *versus* the final structure by some 5°. Using the best-scoring solution as a reference for phase combination within a mean phase difference of 60° leads to a cluster of eight phase sets, which *SHELXE* develops into a full solution after density modification and autotracing, reaching a CC of 38.55% for a main-chain trace comprising 860 residues in 11 chains. As an alternative to *gyre* and *gimble* fragment improvement, using the best-scoring fragments to position the complete original template and subjecting it to LLG pruning leads to comparable starting phases and to an equivalent final solution starting from a single monomer.

#### 3.4.3. Template 4urf   

PDB entry 4urf displays a higher r.m.s.d. over a smaller core than the previous two search models. In this case, correct solutions are found in a single rotation cluster marked by the highest LLG after refinement as well as the highest TFZ score. The best-scoring solution is consistent with two other correct solutions and their phase combination yields a set with a weighted mean phase error of 75°, which develops into a full solution with a CC of 38.0% equivalent to the previous solutions after expansion with *SHELXE*.

### 3.5. Performance of *ARCIMBOLDO_SHREDDER* tests   

This section describes a detailed analysis with the final version of the program for the cases of PPAD and LTG, which were originally solved with a prototype and prompted the development of the *ARCIMBOLDO_SHREDDER* spheres approach. In addition, the α-helical repeat protein (PDB entry 3fp2) and a mixed α/β protein (PDB entry 1yzf) have been selected to test and illustrate parameterization for *ARCIMBOLDO_SHREDDER*.

#### 3.5.1. LTG   

**Figure 4:**
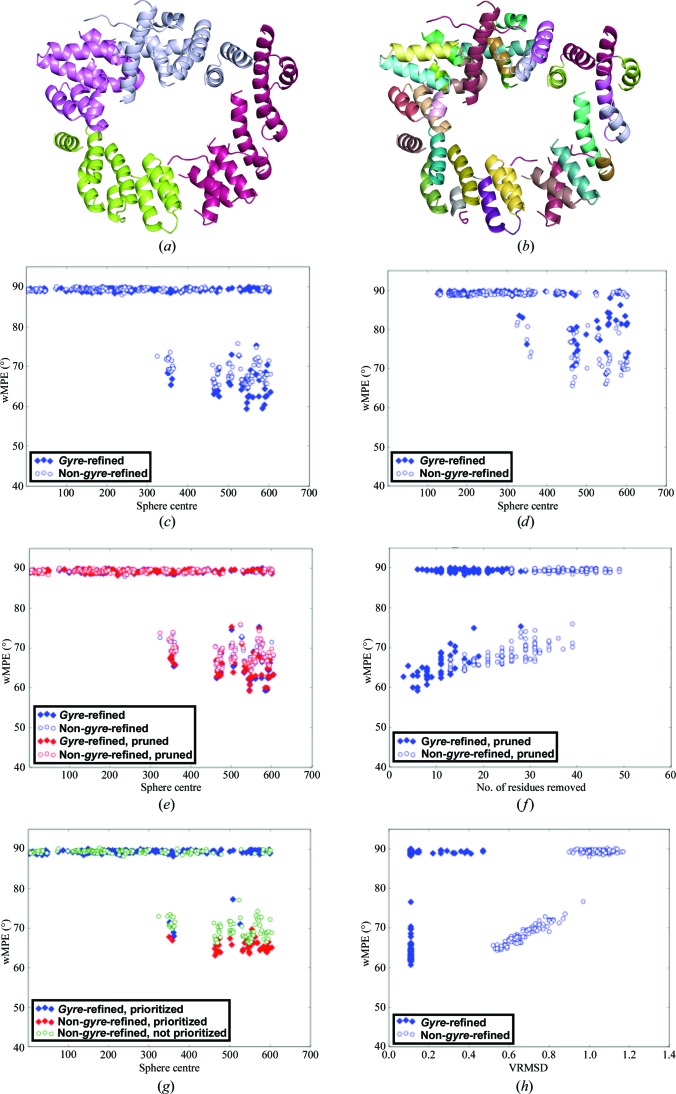
Tests on the LTG structure. Each scatter plot corresponds to a correct rotation cluster. In (*c*), (*d*), (*e*) and (*g*) the horizontal axis represents the number of the central residue of the model. (*a*) First-level annotation groups. (*b*) Second-level groups of helices. (*c*) wMPE *versus* model centre for solutions in *gyre* and *gimble* refinement run 2. (*d*) wMPE for solutions in the run with one cycle of *gyre* refinement at 2.0 Å RMSD (run 5). (*e*) wMPE for all solutions in the run with LLG-based pruning (run 3). (*f*) wMPE against the number of residues trimmed from each solution after LLG-based pruning in run 3. (*g*) wMPE *versus* model centre for solutions in the VRMS refinement run (run 4). A red colour marks solutions that have been prioritized for expansion. (*h*) VRMS against wMPE for all solutions.

In contrast to PPAD, LTG is a highly helical structure (86%) with a low coil fraction. Despite sharing the overall fold, the search template presents an r.m.s.d. *versus* the true structure of 4.6 Å, but helical fragments should be particularly suited for rigid-body refinement, even though the original solution described in §[Sec sec3.2]3.2 was obtained with phase combination of partial solutions before *gyre* and *gimble* refinement were implemented. In addition, many solutions are produced and the effect of parameterization should be more potent than in borderline cases, when solutions are spurious. In particular, eLLG-derived model size, VRMS refinement and LLG-guided pruning as an alternative to *gyre* and *gimble* refinement were probed. In all tests summarized in Table 4 ▸, template annotation and therefore model disassembling were predefined as displayed in Fig. 4 ▸. If *gyre*/*gimble* were performed, a first cycle differentiated four groups in the template, whereas a second cycle would treat each helix as an independent rigid group. Models of 128 or 180 residues were used, corresponding to eLLGs below 30, depending on the RMSD estimation.

*Run 1. Base run without *gyre* or *gimble* refinement*. The *ARCIMBOLDO_SHREDDER* parameterization that best corresponds to the original solution was chosen as a reference. The main difference is that in this test all 417 models generated shared a common size, corresponding to an eLLG of 28 at 1.0 Å RMSD. The selected size would be expected to yield solutions reaching around the inflection point of the LLG sigmoidal curve (McCoy *et al.*, 2017 ▸). Nevertheless, correct solutions of the rotation function become parts of two close clusters populated by more than half of the models, which eventually produce clear discriminated solutions with an LLG well over 60, which is twice as high as in clusters that fail to lead to a solution. 17% of the substructures are nonrandom and the best one develops within *SHELXE* to a main-chain trace of 466 residues and a map with 66° wMPE.
*Run 2. *Gyre* and *gimble* refinement*. The same models were subjected to an initial rotation search and *gyre* refinement at an assumed RMSD of 1.2 Å, distinguishing two rigid groups of the total of four present in the template (Fig. 4 ▸ *a*), followed by 1.0 Å refinement of the rotations and relative translations of each helix in the model (Fig. 4 ▸ *b*). In this case, the initial rotation solutions are divided into the same two close clusters previously seen to contain correct solutions. After both refined and original models were placed, those passing the packing filter were refined with *gimble* subject to the same decomposition as the last *gyre* step.The solution leading to the best polypeptide trace, with a CC of 34.76%, had been processed by *gyre* and *gimble*. The final wMPE, 62°, is decreased *versus* the original run.The graph in Fig. 4 ▸(*c*), displaying all solutions from the main correct rotation cluster, shows how in general *gyre*-refined models improve the wMPE *versus* non-*gyre*-refined models. For correct solutions in this run, the r.m.s.d. between the placed fragments and the LTG structure ranges from 0.3 to 0.45.
*Run 3. Likelihood-based pruning of *gyre*-refined and non-*gyre*-refined solutions*. As an alternative to the *gimble* refinement in the previous run, this run was set to trim incorrect residues using the likelihood-based pruning in *Phaser* (Oeffner *et al.*, 2018 ▸). This refinement is performed for a window size producing a significant change in the eLLG. A threshold in the refined occupancy values for residue trimming is derived by probing different values and choosing the one for which the trimmed model shows the highest LLG. In the present case, model improvement through LLG pruning prior to density modification and autotracing solves the structure as well.Graphs of the solutions for the main correct rotation cluster, identifying them as *gyre*-refined and non-*gyre*-refined and pruned or not, reveal how the best phases correspond to solutions that are *gyre*-refined and trimmed, and how the LLG-based pruning improves the wMPE (Fig. 4 ▸ *e*). Suitably, pruning removes fewer residues from the more correct *gyre*-refined *versus* non-*gyre*-refined solutions, as seen in Fig. 4 ▸(*f*). Pruning of only a few residues can be a good indication of quality, especially for non-*gyre*-refined solutions. Even if phasing is achieved in either case, starting the density-modification step from models containing fewer errors may be beneficial. Some geometrical differences between search model and target, such as backbone torsions, cannot be improved by rigid-body refinement.
*Run 4. VRMS refinement of *gyre*-refined and non-*gyre*-refined solutions*. As models improve upon *gyre* and *gimble* refinement, the r.m.s.d. to the target structure is expected to decrease. This is partially accounted for by decreasing the RMSD value in successive steps, but VRMS refinement in *Phaser* should provide a better estimate of the final r.m.s.d. (Oeffner *et al.*, 2013 ▸), leading to a clearer identification of the solutions to be selected for *SHELXE* expansion.Figs. 4 ▸(*g*) and 4 ▸(*h*) show graphs of the solutions in the major correct rotation cluster. It is noticeable from the plot in Fig. 4 ▸(*h*) that the lowest VRMS corresponds to the best wMPE. VRMS reaches values ranging from 0.11 (for *gyre*-refined solutions) to 0.56 (for non-*gyre*-refined solutions) in correct solutions. The values for the final r.m.s.d. obtained after *gyre* and *gimble* refinement for such correct solutions have indeed improved and range from 0.33 to 0.45 Å.In both the VRMS-refined run and the nonrefined run (run 2) all selected solutions have been *gyre*-refined. Some solutions represented by red diamonds (*gyre*-refined and prioritized) achieve lower starting mean phase errors in the case of the VRMS-refined run (Fig. 4 ▸ *g*).
*Runs 5, 6, 7 and 8. Runs with one cycle of *gyre* refinement and a large starting RMSD parameter*. Finally, four runs were computed with a large initial RMSD to probe whether this could lead to an increase in the radius of convergence in model refinement. A single *gyre* step with a few large groups was undertaken. In run 5, the RMSD was set to 2.0 Å, even though for the same set of models this implies a substantial drop in the eLLG, which becomes 1.7. As in previous runs, close to correct rotations eventually leading to a solution are found in two different clusters, but this time a non-*gyre*-refined solution is the best before expansion and the phases are poorer (wMPE of 66.6°). As can be seen in Fig. 4 ▸(*d*), non-*gyre*-refined models predominate. The *gyre*-refined and non-*gyre*-refined versions of the model are geometrically very similar as only a few large groups have been refined.In run 6, with the same RMSD of 2.0 Å but larger models of 180 alanines, the eLLG increases to a still very modest 3.4. Nevertheless, the number and percentage of correctly placed fragments do not improve compared with the last run and neither do the phases of the placed fragments, corresponding to a wMPE of 67.8° for the best solution, which comes from an original model.Runs 7 and 8 probe the same 127 and 180 alanine models setting the initial RMSD to 3.0 Å and confirm the trend. The smaller models in run 7 altogether fail to produce a correctly phased final structure. Neither refined nor original fragments are placed accurately enough for extension to succeed. Start phases for the few nonrandom solutions are worse by 10° (wMPE of 76.9°) than in previous runs. Again, there is no improvement of refined *versus* nonrefined models. The larger models in run 8 lead to an increase in the number of correctly placed fragments and the start phases they produce improve sufficiently (72.6°) to provide one full solution. In this context, performing the initial refinement of few fragments at high RMSD does not appear to aid convergence, as non-*gyre*-refined models are closer to the true solutions. Accordingly, the program’s default is chosen as 1.2 Å.

In conclusion, for this highly helical model with diffraction data to 2 Å resolution, *gyre* and *gimble* refinement of individual helices improves the models, provided that the RMSD parameter is set to sufficiently low values of around 1 Å. Solutions can be identified by VRMS refinement, while LLG-guided pruning can be also used to trim incorrect fragments and enhances solution.

#### 3.5.2. PPAD   

**Figure 5:**
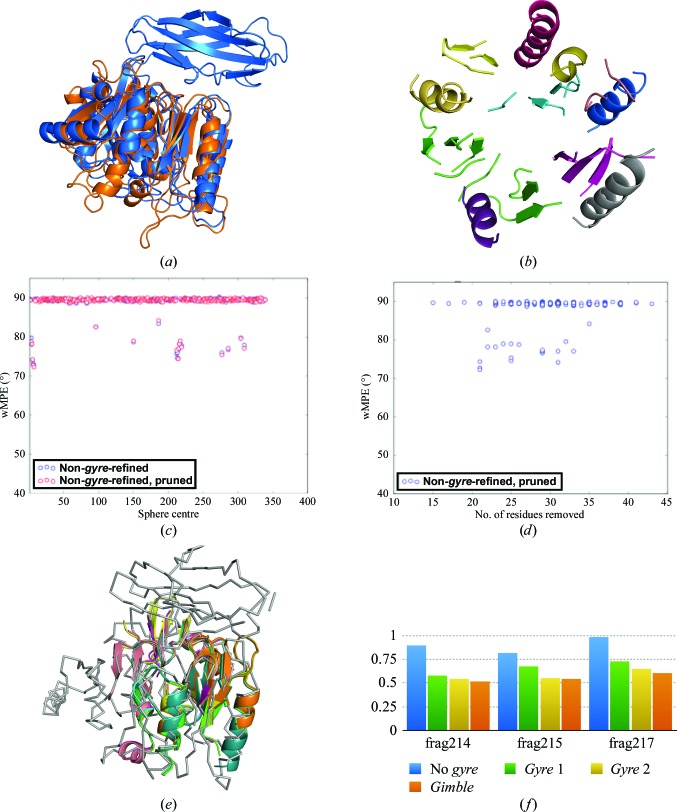
PPAD tests. In runs 1 and 2 coil residues were kept, and run 2 included LLG-guided pruning. In run 3 coil was removed and the models were subjected to *gyre* and *gimble* refinement. (*a*) Superposition between the 1zbr template (orange) and the final structure (blue). The r.m.s.d. is 1.57 Å for a core of 231 C^α^ atoms. (*b*) First level of annotation for the decomposition used in run 3. (*c*) wMPE of solutions *versus* the model centre in run 2. (*d*) Number of residues removed by the LLG-guided pruning against wMPE in run 2. (*e*) The coloured cartoon shows solving fragments from run 2 that clustered together and the grey ribbon shows the final structure. (*f*) R.m.s.d. to the final structure for each of the three correct fragments in run 3. Values at different refinement stages are calculated over a common core.

The final structure of PPAD, superimposed on the template used to solve it, is displayed in Fig. 5 ▸(*a*). PDB entry 1zbr (Northeast Structural Genomics Consortium, unpublished work) shares 19% sequence identity with PPAD and the r.m.s.d. over a core of 231 C^α^ atoms is 1.6 Å. The original solution of this structure (described in §[Sec sec3.3]3.3) involved the combination of two partial solutions from overlapping models derived from PDB entry 1zbr. These models contained 108 and 127 residues, respectively, and had been obtained by preserving coil regions in the starting template. Trimming the coil parts eliminates half of the model, and the resulting fragments fail to produce a solution. The PDB annotates this structure as containing 28% α and 28% β based on *DSSP* (Kabsch & Sander, 1983 ▸). Our automated choice of secondary-structure annotation for *ARCIMBOLDO_SHREDDER* templates is slightly more conservative, leading to a noticeably low secondary-structure content in the case of this template, with 25% α and 33% β, leaving 41% for coil and turns. Considering the large coil fraction in this structure, and the fact that previous successful solution had been accomplished with models preserving it, maintaining coil residues in model generation in *ARCIMBOLDO_SHREDDER* is a choice that may be appropriate in some cases. It must also be considered that the comparatively low fraction of residues in defined secondary-structure elements leads to very fragmented models that are dispersed over a large volume when coil residues are removed. Setting the RMSD to 0.8 Å requires polyalanine models of 101 residues to reach an eLLG of 60. Three runs were compared under such conditions: two of them maintaining the coil regions in the template and one trimming them. In the first two, as the models are continuous, local folds are not disassembled and thus are not given additional degrees of freedom through *gyre* or *gimble* refinement. In the second run, model improvement was attempted within *Phaser* by LLG-guided pruning of residues in the placed model prior to input into *SHELXE*. In the third run, ‘spherical’ search models were generated from the coil-trimmed template and groups of secondary-structure elements (Fig. 5 ▸ *b*) were refined using *gyre* and *gimble* methods. The results of all three runs are summarized in Table 5 ▸.

The first run yields numerous partial solutions within one of the rotation clusters. This is clearly discriminated from all other clusters by its LLG of 64 *versus* less than 30. One of the placed models, the phases of which correspond to a minimum wMPE of 72°, expands to a full solution identifiable by a main-chain trace encompassing 331 residues and characterized by a CC above 30%. The second run is identical to the first, but modifying the models and their selection for density modification and autotracing in the last pruning step. The starting phases are marginally better in some cases (Figs. 5 ▸ *c* and 5 ▸ *d*) and lead to a comparable trace.

Among all placed models in runs 1 and 2 with nonrandom phases only one could be expanded into a full solution. It does not correspond to the top-scoring solution, so the use of phase combination with *ALIXE* was tested to increase the convergence of the method. The solution identified by the top TFZ (7.02) gives rise to a cluster of 14 phase sets gathering solutions with mean phase differences below 60°. Its expansion yielded a trace of 342 residues in 11 chains, characterized by a CC of 37%. All models contributing to this phase cluster are depicted in Fig. 5 ▸(*e*).

No decisive difference is seen by using pruning in terms of number of solutions or figures of merit, but in borderline cases even a slight improvement may help. In general, many residues are being removed (Fig. 5 ▸ *d*), and in this case there is no clear correlation between correct/incorrect solutions and the number of residues removed, even though the solutions with the lowest mean phase error are among those less trimmed.

A third run with less compact models from which coil residues were trimmed, which were subjected to *gyre* and *gimble* refinement, gave rise to fewer but more accurate solutions than the previous runs. Three partial solutions with initial wMPEs of 67.7, 68.7 and 70.8° correspond to models refined with *gyre* and *gimble*. As seen in Fig. 5 ▸(*f*), the r.m.s.d. to the final structure improves in each *gyre* and *gimble* cycle. One of these solutions develops into a full solution that is characterized by a CC of 31.05%.

#### 3.5.3. PDB entry 1yzf   

**Figure 6:**
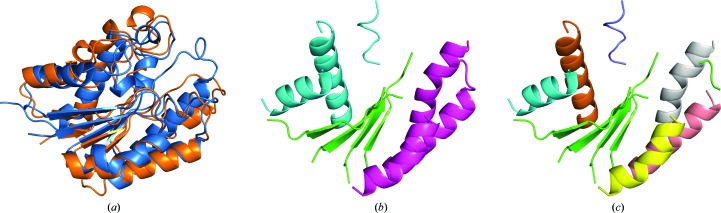
Tests on PDB entry 1yzf. (*a*) Final structure (blue) *versus* the template used in *ARCIMBOLDO_SHREDDER* (orange). The r.m.s.d. between the structures computed with super in *PyMOL* is 2.4 Å over a core of 121 C^α^ atoms. (*b*) Community clustering groups. (*c*) β-Sheet and independent helices grouping.

The *P*3_2_21 crystal form of the lipase/acylhydrolase from *E. faecalis* at 1.9 Å resolution contains a monomer with 195 residues in the asymmetric unit and 36% solvent content. It has a sequence identity of 21% to the homologous esterase EstA from *Pseudoalteromonas* sp. 643A, which was deposited as PDB entry 3hp4 (Brzuszkiewicz *et al.*, 2009 ▸), and an r.m.s.d. of 2.4 Å over 121 atoms (Fig. 6 ▸ *a*).

This case exemplifies a borderline solution owing to the large deviation from the search model, while despite the low solvent content the structure can easily be solved with the same protocols as described but using closer homologues such as PDB entry 4rsh (1.15 Å r.m.s.d. over 116 C^α^ atoms; Midwest Center for Structural Genomics, unpublished work). Secondary-structure annotation of the 185 residues in the 3hp4 template assigned 88 to α-helices and 45 to β-strands. Polyalanine models of 83 residues were generated, corresponding to an eLLG of 60 for an expected RMSD of 0.8 Å. A rotation search and the first cycle of *gyre* refinement (annotation shown in Fig. 6 ▸ *b*) were performed with the RMSD at 1.2 Å, while from the second *gyre* cycle onwards (annotation shown in Fig. 6 ▸ *c*) the rest of the steps were performed at a setting of 0.8 Å. Only one model produced nonrandom solutions. These belonged to rotation cluster 0, one of the four clusters selected by default but containing neither the top LLG scoring solution nor the highest number of models. Among the six correct solutions, the one undergoing *gyre* refinement as well as LLG pruning had the lowest wMPE and better figures of merit. This solution occupies position 51 in the list of 60 substructures prioritized for expansion. Compared with the wMPE of 74° yielded by the unrefined fragment, both the *gyre *and *gimble* or the *gyre* and LLG pruning combinations improve it to 67°. Given the low solvent content, expansion is difficult and a large number of cycles with the latest version of *SHELXE*, featuring constrained autotracing (Usón & Sheldrick, 2018 ▸), are needed to lower the wMPE to 54° and produce an identifiable solution.

An attempt was made to design an improved protocol which would make the solution pathway for this test case more robust. We implemented the possibility of revisiting refinement and/or trimming of the original model. The full, annotated template is superimposed on the solutions that have survived the packing test, whether *gyre*-refined or non-*gyre*-refined. These full models are then rigid-body refined and also subjected to either *gimble* or LLG-guided pruning. In this case, starting from a correctly placed model with high deviations failed to improve on the initial mean phase error, which remained above 72° in spite of the increase in scattering mass, as refinement or trimming did not eliminate the errors sufficiently. Nevertheless, this feature is described as it can be used in the program and may prove useful in other cases.

#### 3.5.4. Tom71 structure (PDB entry 3fp2)   

**Figure 7:**
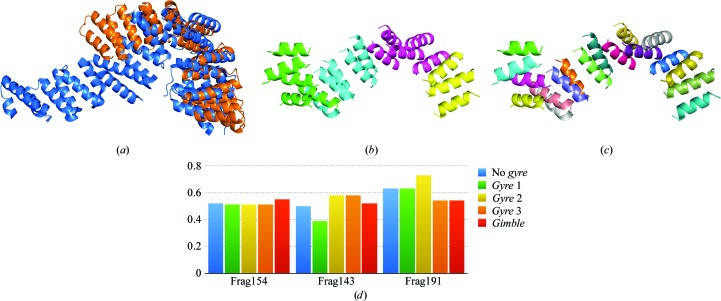
Tests on PDB entry 3fp2. (*a*) Final structure (blue) *versus* the 1w3b template used in *ARCIMBOLDO_SHREDDER* (orange). The r.m.s.d. between the structures is 4.95 Å over a core of 208 C^α^ atoms. (*b*) First level of annotation for refinement. (*c*) Second level of annotation for refinement. (*d*) R.m.s.d. of each of the three correct fragments to the final structure and over a common core using different refinement stages.

Tom71 is a tetratricopeptide repeat (TPR)-containing protein made up of 537 residues comprising 27 helices with 6–22 residues each. TPR domains usually consist of tandem arrays of two antiparallel α-helices that generate a right-handed helical structure. Diffraction data from PDB entry 3fp2 (Li *et al.*, 2009 ▸) extend to a resolution of 1.98 Å. The homologue tested was the superhelical TPR domain of the O-linked GlcNac transferase with PDB code 1w3b (Jínek *et al.*, 2004 ▸), which shares 19% sequence identity with the target structure. Accordingly, the expected RMS (eVRMS) is 1.61 Å, but given the plasticity of the fold both structures can only be partially superimposed. The search model contains 45 helices of 7–14 residues arranged in a fold that locally resembles the target structure through the TPR domains while presenting large overall differences. The superposition displayed in Fig. 7 ▸(*a*) matches 208 residues with an r.m.s.d. of 5.0 Å.

Figs. 7 ▸(*b*) and 7 ▸(*c*) show the template annotation for the first cycle of *gyre* refinement and subsequent refinement steps, respectively. Models with different sizes, comprising three to seven helices each, were tested as well as a range of starting RMSD values from 0.8 to 2.0 Å. The only run that was successful in producing correct solutions was that using the smallest models and the lowest estimated RMSD. In this run, the starting rotation search and first cycle of *gyre* refinement were performed at 0.8 Å RMSD with models of 36 residues corresponding to an eLLG target of 25. Two more cycles of *gyre* refinement were run, decreasing the RMSD to 0.4 Å, which was the value adopted for all remaining steps. Three nonrandom solutions are found among the prioritized solutions, all of them matching models that correspond to arrangements of three helices. The two solutions in the main rotation cluster zero (initial wMPE of 73.4 and 74.5°). Both of them develop to a full solution after density modification and autotracing with *SHELXE* and can be identified by main-chain traces with a CC of 44 and 46%, respectively. A third solution is found in a different rotation cluster (wMPE of 76.6°). It was not sent to expansion as *ARCIMBOLDO_BORGES* stops evaluating clusters once the structure has been solved. The successful models are remarkably small, with barely 5% of the main-chain atoms, but their starting r.m.s.d. to the target structure is already close to 0.5 Å, as seen in Fig. 7 ▸(*d*).

## 4. Concluding remarks   

*ARCIMBOLDO_SHREDDER*, which seeks to improve fragments from distant homologues through refinement against the experimental data, has been extended to derive models of equal size corresponding to volumes representing structural units centred on each amino acid of the template.

The original implementation aimed to leave smaller but more accurate models by identifying and trimming incorrect parts. The present implementation adds the potential to improve the models, progressively subdividing them into rigid structural groups. These are subsequently refined against the rotation function with *gyre* in *Phaser* as well as after placement with *gimble* in *Phaser*. *Phaser*’s LLG-based model pruning may be selected as an alternative to group refinement.

*ARCIMBOLDO_BORGES* is used to evaluate the set of models as a library. Therefore, consistency among partial solutions provides an indication of correctness, which can be further exploited by combining the corresponding phase sets prior to expansion to the full structure with *SHELXE*. Main-chain autotracing in *SHELXE* is used to identify solved structures.

*ARCIMBOLDO_SHREDDER* in spherical mode has been used to solve new and test structures. Its use is intended for challenging cases requiring the improvement of a model from a distant homologue, which on its own does not provide a solution. We have used five different structures to illustrate the features of the program as well as to discuss the appropriate parameterization.

With LTG, a helical case with a large overall r.m.s.d. but where many among the extracted fragments can be correctly placed, we have studied how the convergence of the method can be improved by using *gyre* and *gimble* refinement as well as how VRMS refinement can increase the chances of recognizing correct solutions.

With PPAD, a case with large coil content where rigid-body refinement of individual helices and sheets is of limited use, it was preferable to keep the coil in model generation. This results in more compact models that were best improved through the use of LLG-guided pruning.

With Hhed2, a case with four monomers in the asymmetric unit, we have exploited phase combination of consistent solutions corresponding to the same and different monomers. The first solution of this previously unknown structure involved combination of fragments placed on all four copies.

PDB entry 1yzf is a borderline case that is challenging owing to its low solvent content, where only one model produced nonrandom solutions. Yet, the alternative refinement strategies improved the phases for this solution. This case prompted the development of a protocol to revisit model refinement after translation, superimposing the original template on the possible solutions to restart the refinement of rigid subgroups and trimming.

With PDB entry 3fp2, a large helical structure, we probed a wide range of both the eLLG target and the RMSD used to parameterize the *ARCIMBOLDO_SHREDDER* run, and the results confirmed the low RMSDs required to improve small models.

Current defaults are based on the tests described but should be adapted to the particular case along the lines discussed. Whenever possible, parameterization is set relying on the eLLG, subject to the issue that the r.m.s.d. of the search models produced cannot be reliably estimated. Thus, a pragmatic approach is followed by starting at values of 1.2–1.0 Å, which are high enough to increase the radius of convergence in *gyre* refinement. Refinement steps are iterated, progressively decreasing this value to a final r.m.s.d. of around 0.6 Å as required for successful model expansion through density modification and autotracing.

## Figures and Tables

**Table 1:** X-ray data statistics for all structures used in this study

	PPAD	LTG	Hhed2	1yzf	3fp2
No. of copies in asymmetric unit	1	1	4	1	1
Space group	*P*2_1_2_1_2_1_	*P*6_3_	*P*2_1_2_1_2_1_	*P*3_2_21	*P*2_1_2_1_2_1_
Unit-cell parameters					
*a* (Å)	58.63	163.98	78.02	45.92	47.86
*b* (Å)	60.36	163.98	94.86	45.92	116.29
*c* (Å)	113.88	56.71	140.27	148.03	150.74
α (°)	90	90	90	90	90
β (°)	90	90	90	90	90
γ (°)	90	120	90	120	90
Resolution (Å)	1.5	2.1	1.6	1.9	2.0
〈*I*/σ(*I*)〉	31.62	20.28	12	17.09	39.08
Completeness (%)	99.1	100	100	97.1	95

**Table 2:** Summary of possible operations to modify the search models throughout the program flow

Refinement strategy	Previous step	Next step	Description
*Gyre*	Rotation search	Translation search	Refinement of rigid-body groups against the RF target
*Gimble*	Rigid-body refinement	Density modification and initial correlation coefficient computation	Refinement of rigid-body groups against the TF target
LLG-guided pruning	Rigid-body refinement	Density modification and initial correlation coefficient computation	Trimming of residues from a rototranslated model that upon removal promote an increase of the LLG
Mend after translation	Packing check	*Gimble* refinement	Superposition of the starting trimmed and annotated template over the solutions surviving the packing followed by *gimble* refinement
SHRED-LLG	Rotation search	*ARCIMBOLDO_LITE *with model trimmed according to SHRED-LLG	After rotation search and clustering with the template, systematic removal of residues in different ranges and scoring in a single function for every rotation in order to trim the model of its most incorrect parts

**Table 3:** Summary of the parameterization and the results of the three *ARCIMBOLDO_SHREDDER* runs that led to the successful solution of Hhed2

	Run 1: 4zd6	Run 2: 4z9f	Run 3: 4urf
RMSD (Å)	0.8, 0.5	0.8, 0.5	0.8, 0.5
Model size (No. of residues)	89 (template of 128)	89 (template of 138)	89 (template of 150)
Unique models	95	112	128
Correct solutions	576	19	4
Total solutions	896	1396	1448
Correct ratio	0.64	0.014	0.0027
Lowest wMPE (°)	71.0	73.6	73.9
Top CC for phase cluster (%)	38.0 (starting phase set from combination of three monomers)	38.6 (starting phase set from a single monomer)	38.0 (starting phase set from a single monomer)

**Table 4:** Summary of parameterization and results of the tests performed with the LTG structure

	No *gyre* (reference)	Default	LLG-guided pruning	VRMS refinement	Variation in starting RMSD parameter and model size (runs 5, 6, 7 and 8)			
RMSD (Å)	1.0	1.0, 1.2	1.0, 1.2	1.0, 1.2	2.0	2.0	3.0	3.0
Model size (No. of residues)	128	128	128	128	127	180	127	180
Cycles of *gyre* refinement	0	2	2	2	1	1	1	1
Unique models	417	417	417	417	408	436	408	436
eLLG	28.4	28.4	28.4	28.4	1.7	3.4	0.17	0.34
Correct solutions	205	295	450	296	135	136	5	23
Total solutions	1228	2162	3201	2132	1852	1756	852	1012
Correct ratio	0.17	0.14	0.14	0.14	0.07	0.07	0.006	0.02
Best wMPE (°)	66.3	61.8	61.7	63.1	66.6	67.8	76.9	72.6
Top CC (%)	33.88	34.76	31.84	32.19	30.79	31.39	10.92	32.24

**Table 5:** Summary of the parameterization and the results of the tests performed with the PPAD structure

	Maintain coil	Maintain coil, prune	Remove coil
RMSD (Å)	0.8	0.8	0.8
Model size (No. of residues)	101	101	101
Unique models	335	335	160
eLLG	60	60	60
Correct solutions	32	48	6
Total solutions	1652	2478	1504
Correct ratio	0.019	0.019	0.0039
Best wMPE (°)	72.7	72.1	67.7
Top CC (%)	30.69	31.43	31.05

## Appendix A Implementation and grid computing in *ARCIMBOLDO*

All *ARCIMBOLDO* programs (*ARCIMBOLDO_LITE*, *ARCIMBOLDO_BORGES* and *ARCIMBOLDO_SHREDDER*) are now distributed both through *CCP*4 (Winn *et al.*, 2011 ▸) and as binary files that are available from our web server (http://chango.ibmb.csic.es/ARCIMBOLDO). The same binary executables (Sammito *et al.*, 2015 ▸) run on single machines, either spreading jobs among the available local cores or automatically submitting jobs to be run on a local grid, a remote grid or a supercomputer. They can be started from the *CCP*4*i* interface (Potterton *et al.*, 2003 ▸). *ARCIMBOLDO_SHREDDER* is particularly computationally intensive and will benefit from having access to a supercomputer or grid environment. Implementation of this feature, available in all *ARCIMBOLDO* programs, is described.

The *ARCIMBOLDO* programs support three types of grid connection (local, remote and supercomputer) and some of the more general middleware used in scientific computing, in particular Condor (Tannenbaum *et al.*, 2001 ▸), Sun Grid Engine (Gentzsch, 2001 ▸), Torque/PBS (Staples, 2006 ▸) and some of its variants, such as Slurm, Moab and Maui. Provided a user has access to a grid, configuration is straightforward. Parameters are set in a configuration file (setup.bor) within the template for the particular grid. Mandatory parameters describe the particular middleware implementation, IP addresses, user and queue names if not default, and some configurable choices. This file will be read each time *ARCIMBOLDO* is called and the program will automatically manage all required grid-control and file-transfer operations. While the main program runs on a single workstation, it distributes independent *Phaser* and *SHELXE* jobs to the grid. All control decisions and interpretation of results remains with the workstation. A large number of probe solutions may be generated before a clear discrimination is seen. To prevent the computing setup being loaded with more jobs than it can support, the *ARCIMBOLDO* programs use hard limits on the number of solutions generated at each step. In addition, filters based on figures of merit are used to reduce the number of solutions while preserving diversity. Default limits adapt to the available hardware in the following manner.

(i) If the program is being run in multiprocessing mode, limits and filters will be scaled to the number of available physical cores.
(ii) In any of the grid modes defaults correspond to roughly one order of magnitude more than in the single-workstation case.

As even for a given middleware the setup and configuration vary across sites, choices have been made to provide fail-safe performance. For instance, grid performance is validated as a preliminary check, creating directories, transferring input files if needed, executing *Phaser* and *SHELXE* processes and retrieving the output. During the run, rather than querying queues, output completion and file content is checked. Jobs and their input are packaged in groups, output is retrieved or deleted and so are remote directories after the execution of each given step. Finally, the program has fail-safe implementations in order to handle both normal finishing of the program or a crash owing to an error. In the first case, at each major step of the algorithm (rotation search, translation search *etc.*) summary files are generated and saved in folders that will be recognized if the program is run again in that working directory. This allows changes in the parameterization for particular steps or relaunching an interrupted run without the need to recompute previous steps. In case of a crash owing to an error, the program catches the exception, removes temporary files and exits. After normal termination, no files remain on the remote grid system.
